# Anti-correlations in the degree distribution increase stimulus detection performance in noisy spiking neural networks

**DOI:** 10.1007/s10827-016-0629-1

**Published:** 2016-11-04

**Authors:** Marijn B. Martens, Arthur R. Houweling, Paul H. E. Tiesinga

**Affiliations:** 1 Department of Neuroinformatics, Donders Institute for Brain, Cognition and Behaviour, Radboud University, Nijmegen, The Netherlands; 2 Department of Neuroscience, Erasmus University Medical Center, Rotterdam, Netherlands

**Keywords:** Spiking neural networks, Stability, Sensitivity, Stimulus detection, Degree distribution, Associative plasticity

## Abstract

Neuronal circuits in the rodent barrel cortex are characterized by stable low firing rates. However, recent experiments show that short spike trains elicited by electrical stimulation in single neurons can induce behavioral responses. Hence, the underlying neural networks provide stability against internal fluctuations in the firing rate, while simultaneously making the circuits sensitive to small external perturbations. Here we studied whether stability and sensitivity are affected by the connectivity structure in recurrently connected spiking networks. We found that anti-correlation between the number of afferent (in-degree) and efferent (out-degree) synaptic connections of neurons increases stability against pathological bursting, relative to networks where the degrees were either positively correlated or uncorrelated. In the stable network state, stimulation of a few cells could lead to a detectable change in the firing rate. To quantify the ability of networks to detect the stimulation, we used a receiver operating characteristic (ROC) analysis. For a given level of background noise, networks with anti-correlated degrees displayed the lowest false positive rates, and consequently had the highest stimulus detection performance. We propose that anti-correlation in the degree distribution may be a computational strategy employed by sensory cortices to increase the detectability of external stimuli. We show that networks with anti-correlated degrees can in principle be formed by applying learning rules comprised of a combination of spike-timing dependent plasticity, homeostatic plasticity and pruning to networks with uncorrelated degrees. To test our prediction we suggest a novel experimental method to estimate correlations in the degree distribution.

**Electronic supplementary material:**

The online version of this article (doi:10.1007/s10827-016-0629-1) contains supplementary material, which is available to authorized users.

## Introduction

A fundamental goal of neuroscience is to elucidate how neural circuits respond to small external inputs, while simultaneously remaining stable against neuronal noise. This is especially a problem for cortical networks producing sparse activity, because weak external inputs involve a number of spikes that is comparable to the number of spikes produced by spontaneous activity. Neuronal noise can arise from intrinsic and extrinsic sources and influences every level of the nervous system (Jacobson et al. 2005; Faisal et al. 2008). Noise has in some cases been found to limit the information capacity of neurons (Schneidman et al. 1998; London et al. 2002), but could also enhance the computational capability of neurons in other circumstances (Rudolph and Destexhe 2001; Stacey and Durand 2001).

With the advent of recording and imaging techniques that are not biased to record only from neurons with a high firing rate, experiments revealed sparse firing in the neocortex (Houweling and Brecht 2008; Barth and Poulet 2012; Wolfe et al. 2010). For example, the barrel cortex shows spontaneous spiking at low firing rates, ranging from less than 1 Hz in the superficial layers to a few Hz in the deep layers (Greenberg et al. 2008; de Kock and Sakmann 2009; Barth and Poulet 2012). According to recent experiments, a single extra spike in one neuron in the barrel cortex is amplified and produces approximately 28 additional spikes in its postsynaptic targets, thereby causing a detectable increase in firing rate in the local network (London et al. 2010). The brain thus requires strategies to remain stable against noise in the form of spontaneous spiking activity.

At the same time sensory systems have to be sensitive to relevant external input. Rodents can be trained to use their whiskers to detect an object that predicts a reward and respond with licking to obtain this reward (Huber et al. 2012). The neural responses in barrel cortex to whisker stimulation are hypothesized to play an important role in performing sensory tasks (Petersen and Crochet 2013). Whisker stimulation results in a stimulus-locked neuronal response that can be measured in the rat barrel cortex (Stern et al. 2002). It is even possible to train rats to respond when they detect a small number of spikes elicited by electrical stimulation of a single neuron in the sensory cortex (Houweling and Brecht 2008; Doron et al. 2014).

Thus, neuronal networks need to be stable against intrinsic fluctuations and unrelated spiking input from other brain areas, while the aforementioned experiments showed that these networks are also sensitive to small perturbations. Sensitivity and stability are connected and can in general not be optimized simultaneously, as the increase in one causes a decrease in the other. Increases in sensitivity to external stimuli are mostly studied in terms of modulation of neuronal activity, for example by attention mechanisms (for reviews see Tiesinga et al. 2008; Fries 2009). Here we examine whether specific structures in network connectivity can improve the sensitivity to stability trade-off in spiking neural networks (SNNs). Experimentally, SNNs show spontaneous spiking, which can be amplified through recurrent connectivity into synchronous network-wide activity, referred to as a burst (Martens et al. 2014; Chiappalone et al. 2007). Such bursts can also be evoked in SNNs by external stimulation (Chiappalone et al. 2007). We investigated recurrent SNNs and used simulations to determine the effects of correlation between the number of afferent (in-degree) and efferent (out-degree) connections in neurons on the generation of bursts as part of spontaneous activity and in response to external stimulation. We studied whether stimulation would lead to a detectable change in the firing rate, which in our model would often involve amplification into a burst response. Within the context of our model, a large fraction of the neurons in the network participate in the burst. When comparing to barrel cortex, this core network should be considered embedded in a much larger network. Hence for that case, the network detection corresponds to a smaller fraction of the network becoming active, which is more representative for the experimental situation.

This computational study is the first to focus on the trade-off between sensitivity and stability with correlations between the in- and out-degree in SNNs, rather than in simplified binary networks (Vasquez et al. 2013). The previously studied network of binary neurons contained no inhibition and was captured by a first order Markov process, hence contained no memory of past activity past the current state. The SNNs in this study consist of different neuronal cell types and have connection probabilities representative of cortical networks, and show stable low firing rate and/or brief burst responses, whereas neural networks with binary neurons will converge to either a high or a low firing rate state after a single stimulation (Vasquez et al. 2013). To test network sensitivity we apply nanostimulation (single neuron stimulation) or stimulation of a few neurons (typically four). Our guiding hypothesis is that improved stimulus detection can be achieved through anti-correlations in the degree distribution.

We focus on correlations within the same neurons, rather than degree-correlations between different neurons, which is referred to as assortativity (Newman 2003). Most biological networks are disassortative, such that nodes with many edges preferentially connect to nodes with a few edges (Newman 2003). Assortative networks appear less stable (Brede and Sinha 2005), but at the same time assortative neural networks perform better in detecting subthreshold stimuli and outperform disassortative networks in the case of memory retrieval (de Franciscis et al. 2011; Schmeltzer et al. 2015). Multi-unit recordings in organotypic brain slices suggest a frequency-dependent network architecture, and showed that cortical and hippocampal connectivity is disassortative for low frequencies and cortical connectivity is assortative for the high frequency range in cortex (Ito et al. 2014). These studies thus show that whether high degree neurons preferentially connect to other neurons with low or high degree plays a role in network functioning, and that (dis)assortativity can be found in neuronal networks. However, few studies have focused on correlation in the in-degree and out-degree in the same neurons.

Neuronal network connectivity is not static, but can vary on a timescale of hours (Minerbi et al. 2009) or days (Trachtenberg et al. 2002; Holtmaat et al. 2005), during which synaptic contacts can form and disappear (Yuste and Bonhoeffer 2004). Plasticity has an important role in neuronal circuit formation, in particular in the form of spike-timing dependent plasticity (STDP) which induces competitive learning (Song et al. 2009). We studied networks that were formed randomly (without correlation in the degree distribution) and found that STDP, in combination with a global homeostatic rescaling of synaptic weights, shapes the network such that after pruning the weakest synapses a stable network with anti-correlation degrees is obtained.

When we quantified network stability in the presence of noise, we found that the onset of the high frequency bursting state, a state we consider pathological as noise continuously evokes bursting, was delayed to higher levels of background noise for networks with anti-correlated degrees compared to networks with positive correlations in the degree distribution. Networks with anti-correlated degrees are thus more stable against background noise. We also tested the sensitivity to stimulation for low noise levels, when the networks were not spontaneously bursting, and found that networks with positively correlated degrees were the most sensitive as they produced a burst response for the lowest level of recurrent excitatory connection strength. We then tested stimulus detection, which requires simultaneous stability and sensitivity, by applying stimulation to a few neurons (1-6) under noise levels for which spontaneous network bursts occurred at low rates. The anti-correlated networks outperformed networks with positive correlations. Taken together, these results suggest that the correlation structure is important for the stability and stimulus detection in neuronal networks. Furthermore, we demonstrate that the necessary anti-correlation in the degree distribution can emerge as the result of a simple plasticity rule.

## Materials and methods

In this study, we determine whether correlations in the joint in- and out-degree distribution affect stability, sensitivity and/or stimulus detection performance. We test this in sparsely connected networks of spiking neurons. Here we state the network dynamics and connectivity rules used, and describe how the analyses were performed.

### Network dynamics

The dynamics of the neurons in the model are described by equations proposed by Izhikevich (2003). The Izhikevich model constitutes a simplified version of the Hodgkin-Huxley model. Other appropriate models would be ones whose subthreshold dynamics can be integrated exactly (Rotter and Diesmann 1999), which can be simulated with similar computationally efficient strategies (Yamauchi et al. 2011). For Izhikevich-type neurons, membrane variables *v* and *u* are given as:

1 $$ \frac{dv}{dt} = 0.04 v^{2} + 5 v + 140 - u + I  $$

2 $$ \frac{du}{dt} = a (b v - u) $$

With the following after-spike reset conditions:

3 $$ \text{ if } v \ge \text{ 30, then } \left\{\begin{array}{ll} v \leftarrow c\\ u \leftarrow u + d \end{array}\right.  $$

where the dimensionless variable *v* represents the membrane potential in mV and the dimensionless variable *u* represents the membrane recovery variable, which accounts for the activation of the K ^+^ currents and inactivation of Na ^+^ currents (Izhikevich 2003). The input current I is described in Eq. (4) below. We used the Euler method for integration of the differential equations with smaller integration time steps *dt* (representing milliseconds) than in the aforementioned references in order to increase accuracy, specifically 0.05 ms for the membrane potential and *dt* = 0.1 ms for the other slower variables. The parameters *a*,*b*,*c* and *d* describe the neuronal type, in our model we use the settings for regular spiking (RS), fast spiking (FS) or low-threshold spiking (LTS) model neurons. These parameters are listed in Table 1.

**Table 1:** Parameter settings proposed by Izhikevich to model different neuronal classes found in the cortex (Izhikevich 2003)

Name	Type	N	a	b	c	d	*I*_*fluc*_
Pyr	RS	480	0.02	0.2	-65 ± 5	8	3 ± 0.5
PV	FS	60	0.1	0.2	-65 ± 5	2	0 ± 0.5
Sst	LTS	60	0.02	0.25	-65 ± 5	2	0 ± 0.5

Pyramidal neurons (Pyr) are modeled as regular spiking (RS). The inhibitory population consists of different cell classes: we modeled parvalbumin postive neurons (PV) as fast spiking (FS) and somatostatin positive neurons (Sst) as low-threshold spiking (LTS). ± denotes variance of the underlying normal distribution, representing the variability of parameter values across neurons in the network

The parameter *a*: is the rate of the recovery variable *u*, smaller values result in slower recovery.
The parameter *b*: represents the sensitivity of the recovery variable *u* to the subthreshold fluctuations of the membrane potential *v*, where larger values yield a stronger coupling between *u* and *v*.
The parameter *c*: is the reset value of the membrane potential after a spike.
The parameter *d*: represents the change in recovery variable *u*, caused by spike-activated Na ^+^ and K ^+^ conductances.

We model two sources of noise. The first is the variability associated with small random events, such as ion channel noise and stochastic synaptic release and weak synaptic inputs due to uncorrelated spiking (Jacobson et al. 2005; O’Donnell and van Rossum 2014). These sources of noise contribute only a small fraction to the variability in the input (represented by *I*_*fluc*_ in Eq. (4) below). The other form of noise we simulate is an occasional larger event, such as correlated spiking input events from other brain areas that are unrelated to the sensory stimulus (London et al. 2010), and is referred to as background noise (*I*_*bg*_ in Eq. (4) below). Supplementary Figure ?? shows the flow of current within the network. The cells receive the total input *I* given as:

4 $$ I = I_{fluc} + I_{bg} + I_{stim} + I_{syn}  $$

Where *I*_*fluc*_ is modeled as white noise (for mean and variance see Table 1), and *I*_*bg*_ is modeled as a Poisson process where each background spike event causes a brief current pulse to the excitatory neurons with an amplitude of 15 and a duration of 0.1 ms. The stimulation for our sensitivity measurements is represented by *I*_*stim*_ (parameter settings are given in Section 2.5). *I*_*syn*_ is the conductance-based synaptic input between the recurrently connected neurons, calculated as:

5 $$ I_{syn,j}(t) = \sum\limits_{i} w_{ij} \cdot g_{i}(t) [E_{i,rev} - v_{j}(t)]  $$

**Fig. 1:**
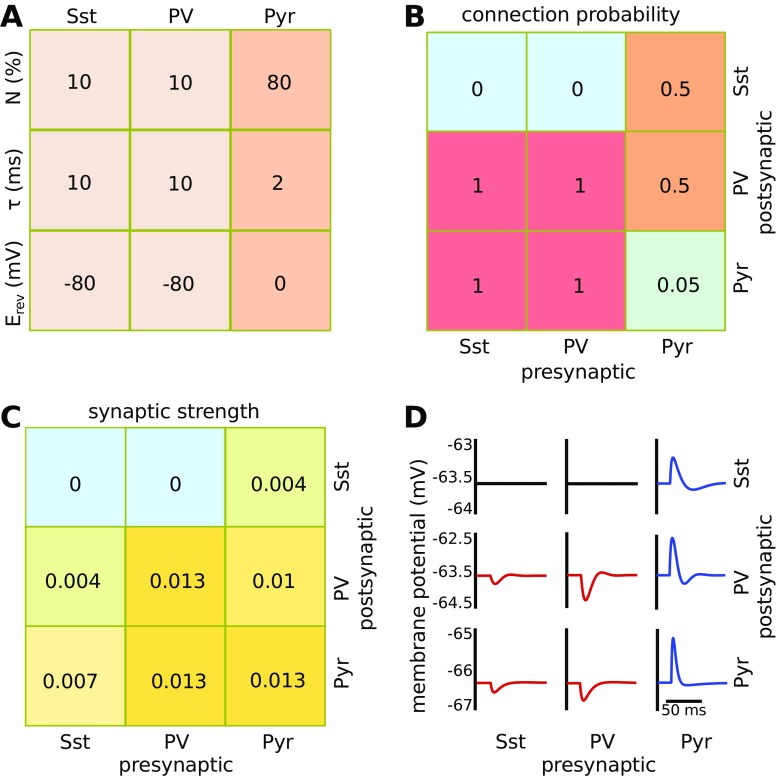
The model network was comprised of one type of excitatory (Pyr) neuron and two inhibitory classes (PV and Sst). **A**: The majority of cells was excitatory and made fast glutamatergic synapses with a reversal potential of 0 (representing mV). The two types of inhibitory neurons projected fast GABAergic synapses with a reversal potential of -80 (representing mV). The synaptic decay constant *τ* depended on the presynaptic neuronal class. Table 1 contains a full description of the neuronal model parameters. **B**: The pyramidal cells have a sparse recurrent connectivity to other pyramidal cells but connect with a high probability to the interneuron populations. In return, both PV and Sst interneurons connected to all Pyr and PV cells, but not to Sst interneurons. **C**: We used the relative connection strength that was found for the inhibitory populations (Pfeffer et al. 2013). **D**: The voltage deflection in response to a single presynaptic action potential when the cells are held at resting potential. The model is conductance based, hence the deflection caused by inhibition is relatively low compared to excitation when the cells are at resting potential

Here *w*_*ij*_ is the synaptic strength between presynaptic neuron *i* and postsynaptic neuron *j*, *g* is the conductance, *E*_*rev*_ the reversal potential for a particular synaptic current (0 for excitatory and -80 for inhibitory neurons) and *v* is the postsynaptic membrane potential. The conductance *g* is increased with 1 for each presynaptic spike and falls off exponentially with a time constant of 2 ms for excitatory, and 10 ms for inhibitory neurons (Fig. 1A).

### Network connectivity

The model network was composed of 600 neurons, of which 80 % were excitatory (pyramidal cells, Pyr) and 20 % were inhibitory neurons. The cortex consists of many functionally distinct inhibitory neuron classes that can be identified by molecular markers (interneuron nomenclature Group 2008; Pfeffer et al. 2013; DeFelipe et al. 2013). Here we used two main inhibitory cell types, namely the fast-spiking parvalbumin-expressing interneurons (PV) and the low threshold somatostatin-expressing interneurons (Sst), (Fig. 1A). The PV cells are critical for the network as they balance the activity of excitatory neurons and stop network bursts from making the network epileptic. The Sst type neurons only get activated for a high level of network activity, and inhibit the PV neurons. These different neuron types are included to accommodate the hypothesis that nanostimulation of inhibitory neurons, which could lead to disinhibition, relates to increased detection performance (see also Buia and Tiesinga 2008). This hypothesis was explored in pilot studies, but was not included in the manuscript.

For a local network of rat neocortical neurons the Pyr-Pyr connection probability is about 5 %, whereas each interneuron projects to most of the local Pyr cells (Holmgren et al. 2003; Packer and Yuste 2011; Pfeffer et al. 2013; Avermann et al. 2012; Lefort et al. 2009), (Fig. 1B). PV neurons are modeled here to receive inhibition from both PV and Sst neurons, whereas Sst neurons only receive excitatory input (Pfeffer et al. 2013; Gibson et al. 1999). The relative fraction of synaptic drive that the interneurons provide is taken from experimental data (Pfeffer et al. 2013) (Fig. 1C-D). This method, proposed by Pfeffer et al., combines a number of measurements in order the determine the strength of the interneuron projection on pyramidal cells as well as on other interneurons. It is important to understand their method in order to appreciate where our parameter settings derive from. First, using paired recordings the probability of a connection between a pre- and postsynaptic neuron (*P*_*con*_) was estimated based on their cell type as well as the unitary strength (*u*_*IPSQ*_) of such connection expressed as the total charge that enters the cell. This is the time-integrated current, and thus represents the product of amplitude and duration. The individual contribution type is then defined as *I* *N* *C* = *u*_*IPSQ*_⋅*P*_*con*_; *INC* thus reports how much inhibition any interneuron of a given class contributes, on average, to any pyramidal cell. The second step is to determine, based on the total charge *I* *P* *S* *Q*_*Pyr*_ entering a pyramidal cell upon stimulation of a particular interneuron population by optogenetic light pulses, how many interneurons (*N*_*inc*_) were activated (and how many spikes per light pulse), i.e. *N*_*inc*_ = *I* *P* *S* *Q*_*Pyr*_/*I* *N* *C*. An interneuron is recorded from simultaneously with the recording of each pyramidal neuron. The interneuron to interneuron strength (*I* *N* *C*_*Int*−*Int*_) can then be estimated using: *I* *N* *C*_*Int*−*Int*_ = *I* *P* *S* *Q*_*Int*_/*N*_*inc*_ (Pfeffer et al. 2013). The strength so measured can be compared and were used as relative strengths in oursimulations.

For many of the connectivity analysis routines, for example to calculate the shortest path length and k-core decomposition, we used the brain connectivitiy toolbox (BCT) (Rubinov and Sporns 2010).

### Correlations in the degree distribution

Our goal is to determine whether correlations in the in- and out-degree distribution are beneficial in that they increase stability and stimulus detection performance relative to uncorrelated networks. We studied the effect of correlations in the degree distribution for the excitatory neurons, whereas interneurons were connected densely but without correlations in the degree distribution (Packer and Yuste 2011). We generated networks from a truncated bivariate Gaussian for the joint in- and out-degree distribution, this allowed the generation of networks with large variance in the in- and out-degree distribution (Vasquez et al. 2013). We start from a bivariate Gaussian with a diagonal covariance matrix given in Eq. (6).

6 $$ p(x,y) = \frac{1}{\sqrt{4 \pi^{2}} \sigma_{x} \sigma_{y}}\cdot e^{\left( -\frac{(x-\mu)^{2}}{2 {\sigma^{2}_{x}}} - \frac{(y-\mu)^{2}}{2{\sigma^{2}_{y}}} \right)}  $$

The bivariate Gaussian can be rotated 45 degrees clockwise or anticlockwise to obtain a distribution with positive (PCOR) and negative (ACOR) correlations, respectively. The mean degree (*μ*) depended on the network size (*N*) and the connection probability (*p*) as *μ* = *N*⋅*p*. The long axis was *σ*_*y*_ = *μ*/3 and the short axis *σ*_*x*_ was set to 0.3⋅*σ*_*y*_. The distributions were truncated at 1 (since a zero degree neuron would not be considered part of the network) and at twice the mean degree to make the distribution symmetric.

Degree distributions were obtained by sampling for each neuron *i*, the in- and out-degree from the corresponding bivariate Gaussian, $d_{i}^{in}$ and $d_{i}^{out}$, respectively. For the uncorrelated control network (UCOR) the list of $d_{i}^{out}$ values was randomly permuted. For the networks with mixed positive and anti-correlations (XCOR), $d_{i}^{in}$ and $d_{i}^{out}$ were sampled for 50 % of the cells from PCOR, and for 50 % of the cells from ACOR distributions. The simplest method for generating a realization of the corresponding network is the configuration method (Newman 2010). A list with $d_{i}^{out}$ stubs for each neuron is made and concatenated into a list $s_{k}^{out}$. Likewise, a list with $d_{i}^{in}$ stubs is made and concatenated into a list $s_{k}^{in}$ and randomly permuted. If the number of out-degree stubs in $d_{i}^{out}$ is larger than the number of in-degree stubs in $d_{i}^{in}$, the lists are ordered and stubs are subtracted starting with the highest out-degrees (one stub per neuron) and added starting with the lowest in-degrees (one stub per neuron) until the lists are matching in number of connections (vice versa for more in-degrees than out-degrees). From these two lists, pairs are picked from the same position, i.e., the *k*th stub on the out-list is matched to the *k*th stub on the in-list to make the connection $s_{k}^{out}$ to $s_{k}^{in}$.

After the initial connectivity was made, we searched for multiple connections between the same pair of neurons and self connections. The overlapping and self connections were mutually permuted using k-permutation (sampling without replacement) using the randperm function in Matlab (The Mathworks, Natick, MA, USA). This procedure was repeated until no overlapping or self connections were found. In the rare case that there was no solution possible, other connections were included in the permutation until we arrived at a connectivity matrix without double or self connections. The probability of obtaining multiconnections were not significantly different between PCOR and ACOR networks (two-sided t-test on *n* = 1000 networks, probabilities are 2.5 ± 0.2 % and 2.5 ± 0.2 %, respectively). However, PCOR networks, which contain neurons with high in- and out-degree, have a significantly higher probability for self-connection than ACOR networks (*p*<0.001 for two-sided t-test on *n* = 1000 networks, probabilities are 0.15 ± 0.04 % and 0.14 ± 0.04 %, respectively). Because overlapping and self connections were mutually permuted, these high in- and out-degree neurons in PCOR networks have a minor bias to preferentially connect to each other due to there being more self connections. However, since we study correlations between in- and out-degree, we prefer to maintain the distribution of the in- and out-degrees compared to, for example, discarding double and self-connections which would lead to a more detrimental bias because more connections will need to be discarded in PCOR networks compared to ACOR networks.

### Network stability

Cortical neuronal networks need to be stable in the sense that stochastic fluctuations should not lead to large increases in the firing rate that could be detected as a stimulation. The stability of the network is quantified in the model by the rate at which background activity triggers synchronous network-wide activity, also called a network burst. To perform burst detection, we used the spike density method (Martens et al. 2014; van Pelt et al. 2004), where a spike density trace is calculated by convolving each spike with Gaussian *G*(*t*).

7 $$ G(t) = A \cdot e^{\frac{-(t-\tau)^{2}}{2\sigma^{2}}}  $$

Where *τ* is the time at which the spike occurred, *A* is the amplitude of the Gaussian (set to 1) and *σ* the width of the Gaussian (2.5 ms).

The start of a burst is defined as the time at which the spike density trace crosses a threshold (10 Hz, which requires about 3 % of the neurons to be active within a 5 ms interval), and the end of the burst is given by the time at which the spike density drops below this threshold.

### Network sensitivity

We tested the sensitivity of cortical neuronal networks to external stimulation. The sensitivity of the network is tested in the model by detecting whether stimulation in a few selected neurons for a fixed duration evokes a network response above a fixed threshold (i.e. 10 Hz); the stimulated neurons were excluded from the burst detection. We selected the stimulated neurons from 10 neurons with an out-degree closest to the average out-degree. Depending on the computer experiment, a number of neurons (*n*_*p*_) were sampled from these 10 neurons. For each stimulation a new set of *n*_*p*_ neurons were sampled. A stimulus input (*I*_*stim*_, Eq. (4)) was applied to the sampled neurons by injection of *I*_*stim*_ = 8 for 25 ms, while the networks were not bursting spontaneously (that is for very low background noise).

### ROC analysis

To produce the receiver-operating curve (ROC), we need to determine the true and false positive rate for a set of detection thresholds. Stimulation was applied every 70 ms. We used a detection window of 60 ms, where we discarded the 5 ms before the stimulation and the 5 ms at the end of the stimulus window. This was performed to avoid the leaking in of the spike density from another stimulus window due to smoothing. We simulated the networks with and without stimulation. A false positive was called when the firing rate exceeded the specified threshold in the unstimulated condition. A true positive was called when the firing rate exceeded the threshold in the stimulated condition. At the start of each stimulus window, all network variables and random number generator seeds were restored to those corresponding to the unstimulated trial; for a fair comparison, the network state and noise at the start of the stimulus trial was thus identical to the stimulus-free trial.

The ROC curve was then obtained by plotting the fraction of false positives against the fraction of true positives for many different thresholds. When there is no effect of the applied stimulus, the number of true positives equals the number of false positives, hence the ROC is the diagonal with an area under the curve (AUC) of 0.5. We tested this protocol by stimulating 0 neurons (i.e. the network behaviour should be exactly the same as for a stimulus-free trial) and found an AUC of exactly 0.5. The deviation of the ROC curves from the diagonal, or equivalently deviation of the AUC from 0.5, is a measure for how different the distributions are and maps for Gaussian distributions on to the effect size of d’, which is the difference in means of the distributions divided by their standard deviation (Kingdom and Prins 2010).

### Plasticity

The number of synaptic connections increases during early development, and subsequent associative plasticity supervises the maturation of cortical circuits, decreasing the number of synaptic connections (Ko et al. 2012; Martens et al. 2015; Johnson 2001). Synaptic stabilization is activity-dependent and involves the formation of PSD-95 (De Roo et al. 2008). PSD-95 is associated with spine stability; weak synapses containing little PSD-95 are in general easily pruned (Holtmaat et al. 2006; Woods et al. 2011).

The number of synapses peaks before the critical rewiring period, and subsequently decreases during further development (Knudsen 2004; Johnson 2001). To mimic the reduction in synapses we initialized UCOR type networks with an excitatory connection probability of 10 %, twice that of the final value of 5 %. The networks were presented with random input in the form of spontaneous release and background spiking (see *I*_*fluc*_ and *I*_*bg*_, respectively in Eq. (4) for details). We applied a spike-timing dependent plasticity (STDP) rule (Song et al. 2009), while the overall level of network activity was maintained by a network homeostasis rule (see below). The simulations were then run for 20 s. The amplitude of STDP was increased and homeostatic plasticity was made faster in order to reduce the length of the simulation period. The results were comparable to those that were obtained for simulations that were run for a longer duration of 50 s. At the end of the simulation the weakest synapses were removed until a connectivity of exactly 5 % remained.

#### Spike-timing dependent plasticity

For the STDP rule we used a function F(Δt) that determined the amount of synaptic modification arising from a single pair of pre- and postsynaptic spikes separated by a time Δt:

8 $$ F({\Delta} t) = \left\{\begin{array}{ll} A_{+} e^{\frac{-{\Delta} t}{\tau_{+}}},& \text{if} {\Delta} t < 0\\ -A_{-} e^{\frac{-{\Delta} t}{\tau_{-}}},& \text{if} {\Delta} t > 0 \end{array}\right.  $$

Where *τ*_+_ = *τ*_−_= 20 ms, $A_{+} = \frac {A_{-}}{1.05} =$ 0.005 (Song et al. 2009). We used a hard upper bound of synaptic strength equal to 0.013. We found that for this synaptic strength neurons fire at rates similar to the target firing rate (Eq. (9)), for the supplied noise level of 0.1 Hz.

#### Network homeostasis

Applying the STDP rule (Eq. (8)) has a strong effect on the postsynaptic firing rate (Song et al. 2009). We therefore maintained the network mean firing rate with:

9 $$ \tau_{h}\frac{dW}{dt}=(R_{tar}-\bar{R})\cdot W  $$

Where W is the connectivity matrix containing the postsynaptic weights of all neurons in the network. According to this rule all synaptic weights in the matrix *W* are adjusted multiplicatively when the current mean firing rate over the last 500 ms ($\bar {R}$) diverges from the target mean firing rate (*R*_*tar*_ = 1.5 Hz); for this process we used a (sped-up) timescale of *τ*_*h*_= 2 s. Experimentally homeostatic plasticity timescales are generally in the range of hours to days (Bateup et al. 2013; Turrigiano 2008).

### Statistical analysis

To test for significant differences between ACOR and PCOR networks we used the 2-sided t-test, implemented as ttest2 in Matlab (The Mathworks, Natick, MA, USA).

We used two methods to test whether correlations in the degree distributions arise when we applied the plasticity rules described above.

For the first method (referred to as the LSR-method) we evaluated the degree correlation using the least squares regression on the in- and out-degree of the neurons; we used the Matlab (The Mathworks, Natick, MA, USA) function polyfit and we tested whether the coefficient of the linear fit was significantly different from a horizontal line (uncorrelated degrees).

For the second method (referred to as the quadrant-method) we plotted the in- and out-degree of the neurons and divided this plot into four quadrants. For the top-right quadrant both the in- and out-degree of the neurons are larger than the mean in- and out-degree, respectively. For the bottom-left quadrant, both the in- and out-degree are smaller than their mean. The number of neurons in these two quadrants (*P*_*n*_) contribute to a positive correlation in the degree distribution. Similarly, the number of neurons in the top-left and bottom-right quadrants (*A*_*n*_) are counted, which contribute to an anti-correlation in the degree distribution. We tested whether ($\frac {P_{n}}{A_{n}}$ - 1) was significantly different from zero using a two-sided t-test.

## Results

### Networks with anti-correlated degrees have the lowest spread in the number of synaptic contacts

**Fig. 2:**
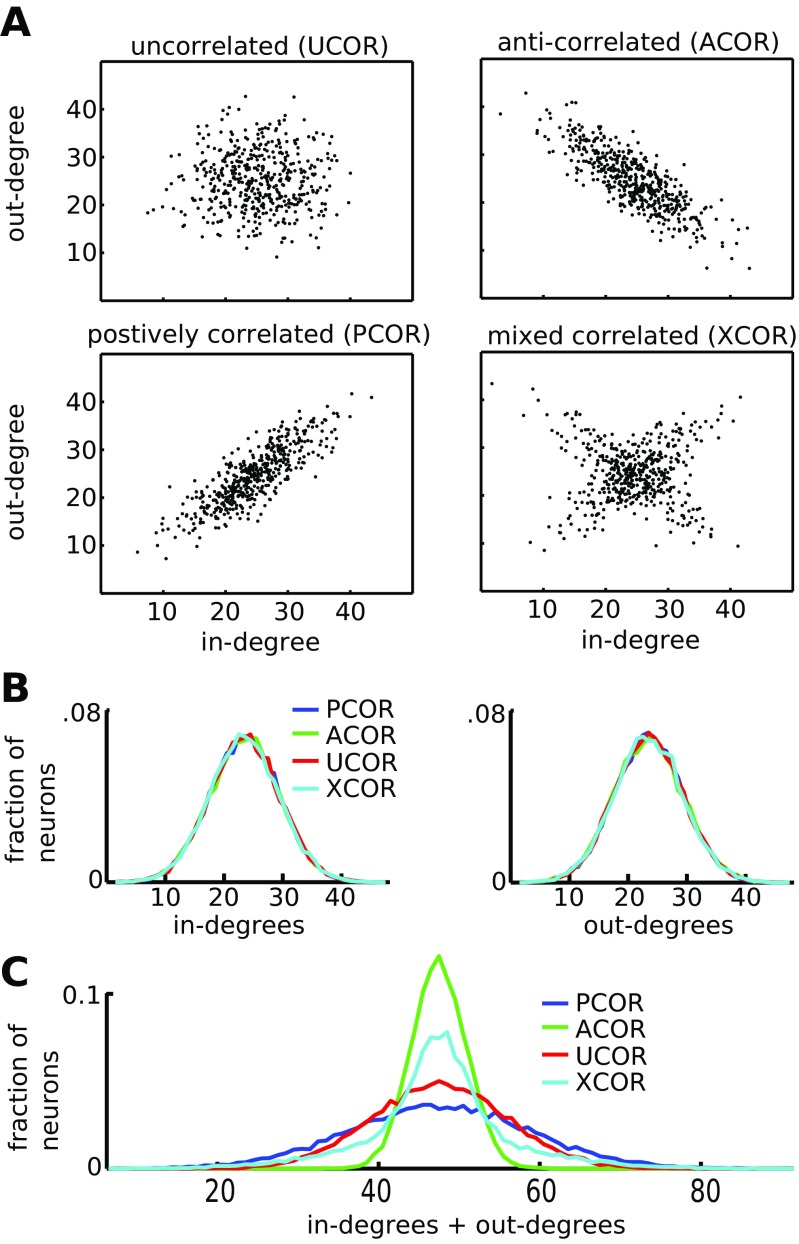
Construction of networks with a correlation between in- and out-degree. **A**: Scatter plots of the in- vs. out-degree for the four network types. The degree distributions were sampled from a truncated bivariate Gaussian, with for each network type a different covariance matrix. For the uncorrelated (UCOR) networks, the covariance matrix was diagonal, with equal variance of the marginal distributions for the in- and out-degrees. To generate correlations we start from a diagonal covariance matrix with unequal variances and rotated it by 45 degrees anticlockwise to obtain anti-correlated (ACOR) networks and by 45 degrees clockwise to obtain positively correlated (PCOR) networks. We also constructed networks where half of the in- and out-degree pairs were picked from an anti-correlated distribution and the other half from a positively correlated distribution (XCOR). **B**: The networks were constructed so that the marginal distributions for the in- and out-degree were the same for the four network types. **C**: The distributions of the sum of in- and out-degree for each neuron shows that ACOR networks have a tight distribution for the total number of connections per cell, whereas PCOR networks show a wider range, with some cells that have few pre- and postsynaptic contacts and others that have many incoming and outgoing synaptic contacts

Here we examined the in- and out-degree distribution of four network types with correlated in- and out-degrees for the neurons: no correlation (UCOR), anti-correlation (ACOR), positive correlation PCOR or a mix of anti- and positive correlation (XCOR, Fig. 2A). The marginal distribution of pre- or postsynaptic connections per neuron is identical for these different networks (Fig. 2B). However, the distribution for the sum of in- and out-degrees shows that ACOR networks have a tight distribution for the sum of pre- and postsynaptic connections per cell, whereas PCOR networks show a wide range of values of the summed degrees, with some cells that make few pre- and postsynaptic contacts and others that have many synaptic contacts (Fig. 2C, in Section 4 we relate these differences to metabolic demands on the cell).

### Networks with anti-correlated degrees have longer path lengths between pairs of neurons and larger structural cores

**Fig. 3:**
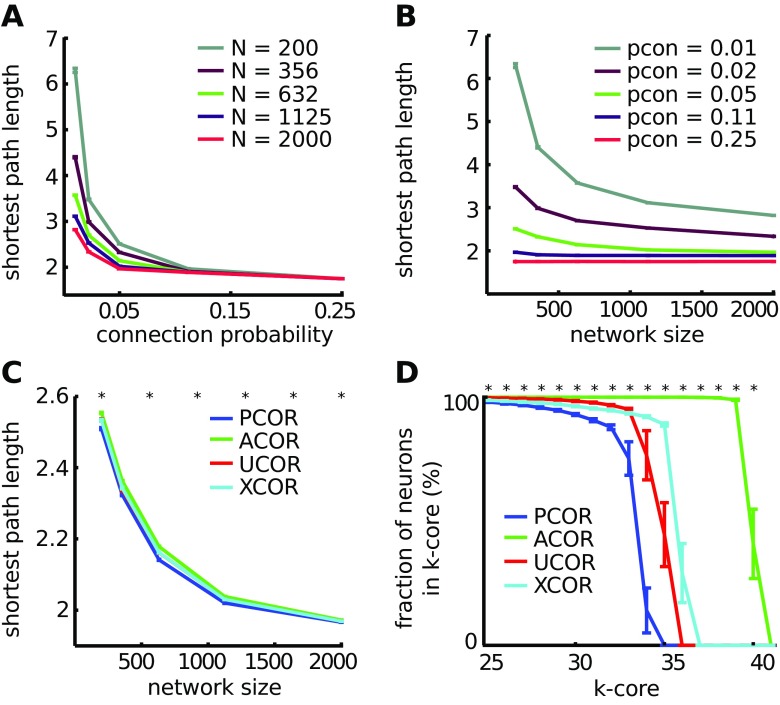
Anti-correlated degrees lead to a higher mean shortest path length between two excitatory neurons and a larger core size. **A**: The shortest path length, which is the mean distance between all pairs in the network, decreases with increasing connection probability. **B**: The shortest path length also decreased with increasing network size. This reduction is most notable for sparsely connected networks (connection probability 0.01). **C**: The four network types were compared for varying network sizes, while the connection probability was fixed to 0.05. ACOR networks have a significantly increased (1-2 %) mean shortest path length. **D**: The results of a k-core decomposition are shown for networks with 480 pyramidal cells and connection probability 0.05. ACOR led to a larger core of highly connected neurons compared to PCOR. Networks in panel A and B were of type PCOR. In all panels, the statistics were averaged across 60 networks for each correlation type, error bars are 1 standard error of the mean (SEM) and stars indicate significant differences between ACOR and PCOR networks according to a two-sided t-test

Having constructed networks with unique correlations in the degree distribution, we wanted to know whether and in what ways the structural connectivity of these networks was different. We used concepts from graph theory that are described in textbooks (Newman 2010). We studied the mean shortest path between the excitatory neurons, which is the shortest path between two nodes, averaged across all pairs and therefore provides a measure of the effective connectivity in the network. Mean path length could be a relevant quantity because it describes how activity can spread across the network to induce a network burst. An increase in connection probability decreased the mean shortest path length (Fig. 3A). By maintaining a constant connection probability and varying the network size, we observed that the mean shortest path also decreases with network size (Fig. 3B). We tested whether the mean shortest path length was affected by correlations in the degree distribution and found that for the typical networks used here (480 excitatory neurons and connection probability 0.05), ACOR networks had a significantly longer mean shortest path length, with an increase of 1-2 % compared to PCOR networks (p < 0.001, significance was tested using a two-sided t-test, Fig. 3C). These differences are small, but become larger for more sparsely connected networks.

Intuitively, a network structural core consists of highly interconnected neurons. To study whether correlations in the degree distribution affected the network structural core size, we performed a k-core analysis (Alvarez-Hamelin et al. 2006). The k-core is the largest subgraph comprised of neurons with a summed in- and out-degree of at least k, which is determined by recursively removing neurons that have a summed in- and out-degree lower than k. At the macroscopic level, when applying k-core decomposition to the connectivity at the level of anatomical brain regions, a structural core remains which is characterized by high metabolic activity that overlaps with the activity in the human brain during the resting state (i.e. the human default mode network), suggesting that a structural core is the basis for shaping brain dynamics (Hagmann et al. 2008). At the microscopic level we found that ACOR resulted in a significantly larger structural core (Fig. 3D).

Taken together, we found graph theoretical differences between the different network types. The number of synaptic connections is more homogeneously distributed in ACOR compared to PCOR networks, which led to a larger structural core size. However, the average shortest path length was increased in ACOR networks compared to PCOR networks. PCOR networks have neurons with high in- and out-degree that function as hubs that reduce the shortest path length. The question is whether these differences have dynamical consequences in terms of stability and sensitivity.

### Networks with anti-correlated degrees are most stable against background noise

In vivo recordings in the rat somatosensory cortex show that cortical neurons fire at a low frequency, ranging from less than 1 Hz in the superficial layers to a few Hz in the deep layers (Greenberg et al. 2008; de Kock and Sakmann 2009; Barth and Poulet 2012). We are primarily interested in the state of low firing rate, in which each neuron is only active a small fraction of the time, because this state allows a stimulation to cause a detectable difference in the network firing rate. We therefore quantified the stability of each of the four network types (see Fig. 2A).

**Fig. 4:**
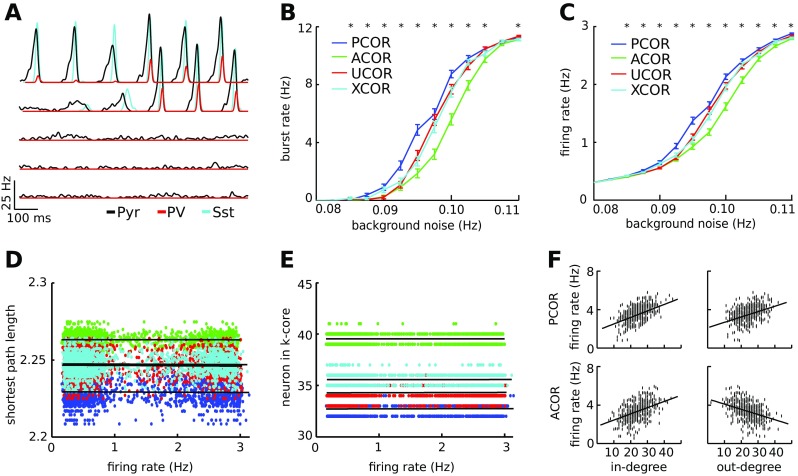
Anti-correlation in the degree distribution increases stability against noise. **A**: Averaged firing rates of excitatory (black lines), PV (red traces) and Sst (cyan traces) neurons in response to background noise events at frequencies between 0.08 Hz and 0.1 Hz per neuron. The low noise input evokes background spiking activity without bursting (lower traces), whereas the high noise input rates trigger periodic synchronized bursting activity in the network (upper traces). **B**: The PCOR networks (blue) produce network burst activity for a lower noise rate than the ACOR networks (green). UCOR (red) and XCOR (cyan) correlated networks showed intermediate levels of stability. **C**: As expected for a lower burst rate, the ACOR networks also have a lower firing rate compared to the PCOR network for an equal amount of random input spikes. **D**: The ACOR networks have on average a longer shortest path length (also see Fig. 3). The shortest path length for a given network was not correlated with the firing rate in that network, as the Pearson correlation value was not significantly different from zero (p > 0.05). Each dot represents the mean firing rate of the excitatory network; background noise rates varied between 0.075 and 0.11 Hz. **E**: No correlation was found between the mean firing rate of a network and the largest k-core in that network. Statistics and color convention were as in panel D. **F**: Dots represent the firing rate of a single neuron plotted against its in- and out-degree for one network in the bursting state (input rate 0.11 Hz per neuron). In panels B to E the statistics are averaged across 120 networks, error bars are 1 SEM and stars indicate significant differences between ACOR and PCOR networks according to a two-sided t-test. Each network consisted of 480 pyramidal, 60 PV and 60 Sst neurons

In the absence of noise, no spiking activity was detected in any of the networks. For a low level of background noise, the networks remained stable and fired irregularly, while increased noise results in unstable, continuous network bursting (Fig. 4A). The excitatory synaptic strengths were such that low frequency spiking input could evoke a detectable response.

We found that ACOR networks showed fewer burst responses for the same level of noise compared to PCOR networks (Fig. 4B). This also related to a lower firing rate in ACOR networks (Fig. 4C). Thus PCOR networks were less stable than ACOR networks. Stability for UCOR and XCOR networks was in-between ACOR and PCOR networks.

We wanted to know whether these differences in stability could be explained by the different graph theoretical properties found above. For a given path length the properties such as firing rate were broadly distributed, but there was no statistically significant trend observable between the mean firing rate of the network and the mean shortest path length of the associated network (Pearson correlation values were not significantly different from zero, Fig. 4D). We also did not find a correlation between k-core size and firing rate (Fig. 4E). Thus, for the same number of connections in a network, the mean pair distance and structural core size did not influence the network stability.

We then studied the relation between in-degree and firing rate for individual neurons. A high in-degree led to a high firing rate (Fig. 4F). Given the correlation structure in the network, this means that high out-degree neurons in a PCOR network have high firing rates, whereas the high out-degree neurons in the ACOR network have low firing rates. Hence, the anti-correlated degrees directly result in a reduced synaptic output to the network in reponse to noise, providing an intuitive understanding of the mechanism by which the additional stability is generated.

### Positively correlated networks are most sensitive to stimulation in the absence of spontaneous bursts

**Fig. 5:**
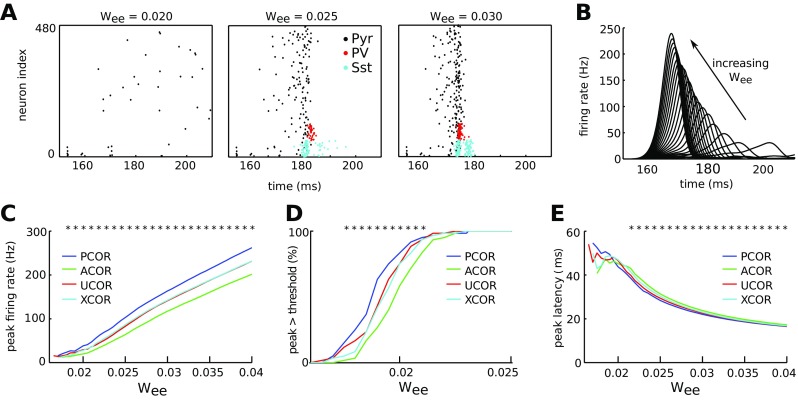
Networks with positively correlated degrees are more sensitive to a small perturbation for the low noise condition, for which there are no spontaneous bursts. **A**: Rastergrams wherein each dot represents a spike. The spikes of excitatory pyramidal cells are in black, PV interneuron spikes are in red and Sst interneurons spikes are in cyan. Depending on the strength of recurrent excitation (*w*_*ee*_), external stimulation in 6 neurons of an UCOR network leads to either a weak response of varying duration that did not recruit inhibitory neurons, or a strong, sharp response that recruited inhibitory neuron activity that curtailed the burst. Each neuron received input from background spikes at a rate of 0.07 Hz. Interneurons were recruited only when a network burst occurred. The rastergrams also show the spikes of the stimulated neurons, but these were not included for the burst detection and post-stimulus time histograms to avoid stimulation artifacts. **B**: The smoothed post-stimulus time histogram of excitatory neurons for 25 different values of the recurrent excitatory strength, equally spaced between 0.015 and 0.04. For smoothing see Eq. (6). **C**: Mean peak firing rate in the smoothed post-stimulus time histogram plotted against the recurrent excitatory strength. PCOR networks (blue) showed higher peak firing rates compared to the ACOR networks (green) for equal recurrent strength. **D**: Bursts were detected when the recurrent strength exceeded 0.015, and for recurrent strength 0.023 and higher the network consistently showed a burst response after each stimulation. **E**: The peak latency, which is the time between the onset of stimulation and the peak of the burst, decreased for stronger recurrent strength. For recurrent strength below 0.02, variability in the peak latency is high due to the low number of detected bursts. For each network type the statistics are averaged across 60 networks with one stimulation per network, error bars are 1 SEM and stars indicate significant differences between ACOR and PCOR networks according to a two-sided t-test

Whisker stimulation results in time-locked responses that can be measured in the rat somatosensory cortex (Stern et al. 2002). These neuronal responses are hypothesized to play an important role in performing and learning sensory tasks (Huber et al. 2012; Petersen and Crochet 2013). Rats are better at detecting external stimulation when multiple neurons are activated compared to when a single neuron is stimulated (Romo et al. 1998; Houweling and Brecht 2008). Here we studied the network responses upon stimulation of 6 neurons while the networks were not bursting spontaneously (0.07 Hz background noise). For weak excitatory coupling strength, only a few neurons in the network responded to stimulation in addition to the directly stimulated cells (Fig. 5A). Neuronal recruitment increased with excitatory coupling strength (Fig. 5B), where PCOR networks had a higher peak firing rate than ACOR networks (Fig. 5C). Network-wide burst responses, detected when the firing rate crossed a predefined threshold, were realized for weaker coupling strengths in the case of PCOR networks (Fig. 5D), and these networks were fastest to reach their peak activity (Fig. 5E). Taken together, these data show that networks with positive correlations in the degree distribution, in the absence of spontaneous network bursting, are most sensitive to stimulation of a few neurons.

### Stimulus detection is enhanced in anti-correlated networks for higher background noise

**Fig. 6:**
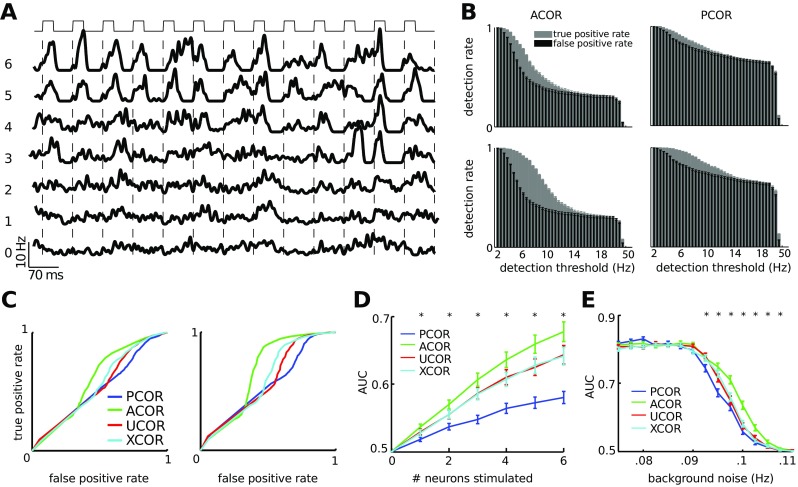
Stimulus detectability, as evaluated by ROC analysis, is higher for networks with anti-correlations in the degree distributions than for networks with positive degree correlation. **A**: Spike density for the excitatory neurons in a stimulated ACOR network. Stimulation was applied every 70 ms to up to 6 neurons in the network; the stimulus duration is indicated by the block pattern (top). At the start of each stimulation, which is indicated by dotted line, the network variables were reset to their baseline value (no stimulation, bottom). Under the same noise conditions as the baseline trace, the number of stimulated neurons increased from bottom to top with a step of one. In the top trace stimulation was applied to 6 neurons, which often initiated a detectable response including many neurons. Background noise was 0.098 Hz per neuron. **B**: Stimulation of 3 (top) and 6 (bottom) neurons was applied to ACOR (left) and PCOR networks (right), while the background noise was identical. Stimulation in the ACOR networks was better detectable than stimulation in the PCOR networks because the PCOR networks were less stable to noise. **C**: The corresponding ROC curves for 3 (left) and 6 (right) stimulated neurons quantify the stimulus detectability, such that curves further away from the diagonal relate to higher detection rates. Because neuronal networks respond non-linearly to noise, which occasionally initiated bursting in response to spontaneous activity, additional stimulation in that case did not increase the response amplitude further, therefore the bottom-left part of the ROC curves remain along the diagonal. **D**: The area-under-curve (AUC) of the ROC is a quantification of stimulus detectability. Detectability increases with the number of stimulated neurons and is highest for ACOR networks. **E**: We stimulated four neurons under different background noise levels. ACOR networks showed higher detectability in the high noise conditions compared to PCOR networks. For each correlation type the statistics are averaged across 120 networks, error bars are 1 SEM and stars indicate significant differences between ACOR and PCOR networks according to a two-sided t-test

We showed that in the absence of spontaneous bursting the PCOR networks were most sensitive to stimulation, whereas ACOR networks were found to be more stable against noise. Here we investigate the sensitivity to external stimulation for varying degrees of background noise; this provides a direct quantification of detection performance. For our experiments we first supply the networks with background noise in the form of random spiking in each of the excitatory neurons of the network (see Methods, Section 2.5). We then applied stimulation in 1 to 6 neurons and observed a moderate to clearly noticable increase in the firing rate. (Fig. 6A).

We used these experiments to study whether stimulation had a detectable effect on the network activity (Fig. 6B). For low detection thresholds, detection of both true and false positive network events is high. For intermediate detection thresholds we observed that ACOR had lower rates of false positive events compared to PCOR networks (Fig. 6B). From the true and false positive rates we constructed ROC curves (Fig. 6C). From these ROC curves we extracted the area under the curve (AUC) as a measure of stimulus detection in noisy conditions, and show that for stimulation of a few (1 - 6) neurons stimulus detection in ACOR networks was enhanced compared to PCOR networks (Fig. 6D). Nanostimulation (single neuron) had a small but significant effect on stimulus detection. We then studied the stimulus detection under varying background noise levels (Fig. 6E). We found that ACOR networks were able to detect stimuli for stronger background noise, which can be attributed to the increased stability against background noise as shown before (Fig. 4B-C).

### Effect size depended on connection probability and network size

We showed that ACOR networks outperform PCOR networks in detection of external stimulation under high levels of noise. Next we wondered what influence connection probability and network size had on stimulus detection. For these simulations we maintained constant synaptic strengths. We observed that for a high connection probability (10 %) network bursting occurred at noise levels around 0.07 Hz, whereas networks with a low connection probability (1 %) did not burst until noise levels reached rates around 0.14 Hz (Supplementary Figure ??). For connection probability 5 % and 10 % our previous results that ACOR outperforms PCOR were confirmed. When connection probability was lowered to 3 % and below, the stimulation of a few neurons was difficult to detect and the advantage of ACOR to outperform PCOR disappeared. When connection probability was further reduced to 1 %, the ability to detect external stimulation was almost completely abolished. We attribute these findings to the higher mean out-degree in the densely connected networks compared to sparsely connected networks, thereby allowing external stimulation of a few neurons to recruit a larger synaptic drive to the rest of the network. For our stimulation protocol involving 600 neurons, stimulation in four neurons and our setting of synaptic strength, the minimal connection probability to detect external stimulation was ∼5 %.

Furthermore, we varied the network size and observed that for larger networks the effect size by which ACOR networks outperformed PCOR networks in terms of sensitivity to nanostimulation was increased (Supplementary Figure ??). Additionally, we studied the influence of the effective time step used for the numerical integration and found that the results were robust when we used a smaller time step (dt=0.02 ms). Furthermore, using an identical time step for *u* as the other variables (see Methods for details), also showed consistent results (Supplementary Figure ??).

### Associative plasticity forms anti-correlations in the degree distribution

How could networks with anti-correlations in the degree distribution emerge? Several different models exist for the establishment of synaptic connections, but these do not take into account correlations in the degree distribution (Yoshihara et al. 2009; García-López et al. 2010). We studied whether correlations in the degree distribution could emerge from associative plasticity.

Early in development the number of synaptic connections is high, and subsequent associative plasticity reorganizes the cortical circuits, decreasing the number of synaptic connections (see Section 2.7 for details). We constructed networks with 10 % connection probability to represent the more densely connected networks early in development. These networks were of the UCOR type to mimic the random organization. Uncorrelated spontaneous spiking was supplied to the network as synaptic inputs with an amplitude of *I*_*bg*_ = 15, duration of 0.1 ms and at a rate of 0.1 Hz for each neuron. The rate of 0.1 Hz was chosen because the ACOR networks that were generated from a bivariate Gaussian distribution then fired at ∼1.5 Hz, for which the occasional synchronized burst emerged. When in these networks the synaptic strength was modified by STDP and the set point rate for the homeostatic process was set to 1.5 Hz, spiking activity still propagated throughout the network accompanied by the occasional synchronized network burst.

**Fig. 7:**
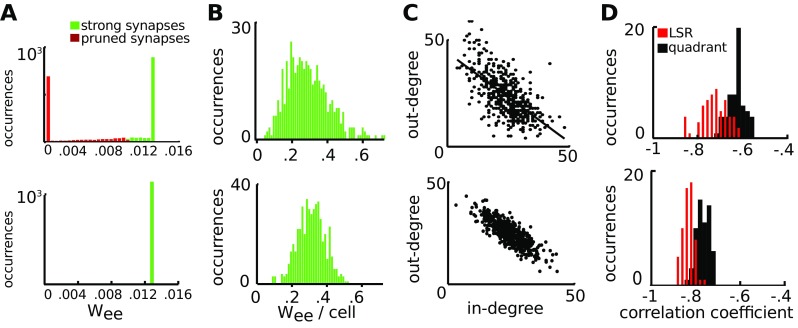
Associative plasticity forms networks with anti-correlation in the degree distribution. **A**: Top: an UCOR network with a connection probability of 10 % with an upper bound on synaptic strength of 0.013 was run for 20 seconds with spike-timing dependent plasticity. The amplitude of STDP was increased and timescale of homeostatic plasticity was decreased compared to their values in the literature in order to reduce the duration of the simulation. At the end of the simulation period the synaptic distribution was bimodal. The weak synapses (red) were pruned and removed from the distribution until a connectivity of exactly 5 % was obtained. The synaptic strength of the remaining synapses (green bars) was comparable to an ACOR network explicitly constructed from a bivariate Gaussian distribution (bottom). **B**: The summed excitatory synaptic strength for the STDP-generated network (top) and the explicitly constructed ACOR network (bottom). **C**: In- and out-degrees of the STDP-generated network (top) and the explicitly constructed ACOR network (bottom). Black line is fitted using the LSR-method. **D**: 60 STDP-generated networks (top) and 60 explicitly constructed ACOR networks (bottom) were tested for correlations in the degree distribution using the LSR-method and the quadrant-method (see Materials and Methods). All STDP-generated networks showed anti-correlations in the degree distribution that were comparable to the generated ACOR networks. The LSR-method shows that the angle of the slope is similar for the explicitly generated and STDP-generated networks, whereas the lower values found for the STDP-generated networks using the quadrant-method are due to the increased variance across independent realizations

We observed that associative plasticity reorganized the synaptic weight distribution towards a bimodal distribution (Fig. 7A). Synapses were pruned (removed), starting with the weakest synapses, until a connectivity of 5 % was obtained (Fig. 7A). We summed the synaptic inputs to each of the neurons and found that the distribution was comparable to explicitly constructed ACOR networks from a correlated bivariate Gaussian distribution (Fig. 7B).

By plotting the in- and out-degree of the synaptic connections that remained after associative plasticity, we observed anti-correlation in the degree distribution (Fig. 7C). We calculated the correlation (Section 2.8) for 60 networks with randomly initialized dynamics and connectivity, and found that anti-correlation in the degree distribution was consistently formed (Fig. 7D).

Consistent with the bimodal STDP rule, we found that applying a weight-dependent STDP rule, as described by Morrison et al. (2007), to UCOR networks with 10 % connectivity, resulted in networks for which the 5 % strongest synapses have an ACOR distribution (Supplementary Figure ??).

In summary, for these parameter settings, dense and uncorrelated networks were consistently reorganized into more sparsely connected networks with anti-correlation in the degree distribution by synaptic pruning.

## Discussion

The activity produced by cortical microcircuits in sensory areas provides the opportunity to detect external stimuli, provided that the circuits are stable against noise generated by spontaneous firing. Such simultaneous sensitivity and stability is difficult to achieve (Vasquez et al. 2013). Previously, in a simple recurrent network of stochastic binary neurons, it was numerically shown that stability was increased for ACOR relative to PCOR networks. Nevertheless, these ACOR networks consisting of binary neurons had the same level of sensitivity compared to PCOR (Vasquez et al. 2013).

Here we studied the effects of correlation between in- and out-degree on stimulus detection in recurrent spiking neuronal networks. We found that ACOR networks had increased network stability, whereas in our simulations of the low noise state, without the spontaneous bursting activity, sensitivity was highest for PCOR networks. The rat somatosensory cortex shows spontaneous spiking at firing rates of up to a few Hz (Greenberg et al. 2008; de Kock and Sakmann 2009; Barth and Poulet 2012). When we performed stimulation in the more realistic setting of spontaneous background spiking, representative of these experimentally observed network states, we found that detection performance was highest for the ACOR networks. High noise levels bring the recurrent networks to a pathological bursting regime, with high frequency spontaneous bursting which results in a high false positive rate. Anti-correlations in the degree distribution provide stability to the network, and as a consequence a lower false positive rate. At the same time, these ACOR networks remain sensitive to external stimulation, thus simultaneously improving stability and stimulus detection compared to PCOR networks. Our hypothesis is that stimulation detection corresponds to a nonlinear increase in neural activity in sensory areas. In our model, we use bursts as a proxy for such an event. As our model networks represent only a small part of the entire barrel cortex network, the bursts correspond to a more modest increase in the barrel cortex activity. Specifically, they should be experimentally observable as a modestly increased rate coupled to a strongly increased level of synchronization in sparsely active networks (Houweling and Brecht 2008; Barth and Poulet 2012; Wolfe et al. 2010). We further speculate that downstream neurons in areas that plan actions (i.e. the initiation of licking) have become more sensitive to these synchronously active neurons, for instance, through a Hebbian mechanism during training.

By dissecting the firing rate based on in-degree, we found that in the ACOR networks the neurons with high out-degrees had on average a lower firing rate; this effectively reduces the excitatory input to the network during spontaneous activity. Concurrently, the high in-degree neurons collect inputs from many neurons in the network, and have a higher than average firing rate, but project their output to a relatively small portion of the neurons so as to not destabilize the network. As a consequence, stimulating the average out-degree neurons in the ACOR networks results in a burst response even though the networks remained more stable against the noise-induced bursts compared to the PCOR networks. These findings provide an intuitive understanding of the mechanism by which the ACOR networks were more stable to noise than PCOR networks, which improved the stimulus detection performance in ACOR networks. It was recently shown *in vivo* that network firing patterns are largely dictated by basic circuit variables (Okun et al. 2015; Harris and Mrsic-Flogel 2013). We suggest correlations in the degree distribution contribute as a basic network property to the maintenance of stable spiking activity in neuronal networks.

We investigated the stability and sensitivity in spiking neurons, where, due to the presence of inhibitory neurons, the network can be in a regime with no or a few bursts during spontaneous activity, and brief bursts terminated by recruited inhibition can be induced by electrical stimulation. Key relevant features of the neuron dynamics are integration of multiple synaptic inputs into an output spike, a refractory period as well as an effective inhibitory feedback. These features are also present in other spiking neuronal models, i.e. LIF neurons and multicompartmental models with Hodgkin-Huxley currents, and when properly parameterized we expect similar results. The results will of course be different, when, in the latter, the model neurons can switch between spiking and (single neuron) bursting states, as this will make induction of a network burst easier and less dependent on network structure, and when the integration properties are different, i.e. higher sensitivity for inputs with a certain values for inter-input intervals, for example for the resonate-and-fire neuron proposed by Izhikevich (Izhikevich 2001).

For the neuronal networks in this study we found that sensitivity to stimulation of a few neurons requires a minimal connection probability. The effect size by which ACOR networks outperformed PCOR networks increased with connection probability and network size. Although many synaptic connectivity features are ubiquitous among cortical system, experimentally observed connectivities differ between species and sensory modality (for review see (Chapeton et al. 2012)). It is interesting whether the ability to detect nanostimulation is, for example, different between rats and mice, and whether visual, auditory and somatosensory regions show a difference in detection performance. We predict that densely connected regions show better performance compared to sparsely connected regions. Inhibitory neurons are less abundant in cortical circuits than excitatory neurons, but are more densely connected to the excitatory population (Pfeffer et al. 2013). Nanostimulation of inhibitory neurons might therefore have an increased detection performance compared to nanostimulation of excitatory neurons.

**Fig. 8:**
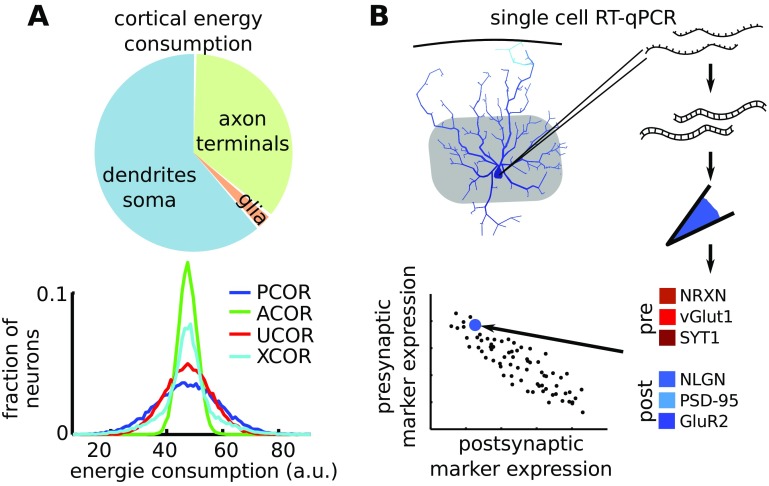
Metabolic consequences of, and a method to experimentally confirm, anti-correlated degrees in the cortex. **A**: The pre- and postsynaptic parts of the neuron have a comparable energy consumption ((Harris et al. 2012; Attwell and Laughlin 2001), estimates in the diagram are from (Harris et al. 2012)). By assigning equal levels of energy consumption to the pre- and postsynaptic part of each synapse, we find that energy demands are more homogeneously distributed over cells in ACOR networks compared to PCOR networks. **B**: To estimate the in- and out-degree of single neurons, we propose to use single cell RT-qPCR and compare the relative expression of pre- and postsynaptic markers. This can for example be performed for RNA encoding presynaptic proteins Neurolexin (NRXN), Vesicular glutamate transporter 1 (vGlut1) and Synaptotagmin-1 (SYT1) (Sudhof 2008; Beaudoin et al. 2012; Tang et al. 2006); and postsynaptic proteins Neuroligin (NRGN), postsynaptic density-95 (PSD-95) and Glutamate receptor 2 (GluR2) (Sudhof 2008; Beaudoin et al. 2012; Dingledine et al. 1999)

Synaptic communication places a disproportionally high demand on energy consumption (Mink et al. 1981; Harris et al. 2012). Pre- and postsynaptic parts of the neuron consume a comparable amount of energy (Attwell and Laughlin 2001; Harris et al. 2012). Cells that are stressed by excessive ATP consumption can produce damaging levels of reactive oxygen and nitrogen species (ROS/RNS) in the cell, leading to protein dysfunction and potential cell death (for review see (Wang and Michaelis 2010)). Cell death by oxidative stress is linked to neurodegenerative diseases (Wang and Michaelis 2010). When networks have anti-correlations in the degree distribution, the energy demand is more homogeneously distributed over the neurons (Fig. 8A). Thus, by making cellular demands on energy consumption more homogeneous, the anti-correlation in the degree distribution provides another level of robustness to brain networks.

For standard growth models the number of pre- and postsynaptic connections for each neuron is set to be independent, and there is no correlation in the in- and out-degrees. These networks will be of type UCOR. Previously, STDP was shown to lead to non-random structures (Kato and Ikeguchi 2010; Masuda and Kori 2007) and disassortivity in network connectivity (Kato et al. 2009). These authors also studied the distribution of in-degree, out-degree and sum of in- and out-degree. They observed a general reduction in the out-degree, particularly for neurons with high in-degrees, but did not quantify the correlations in the degree distribution (Kato et al. 2009). We demonstrated that STDP can reorganize UCOR networks into networks with anti-correlation in the degree distribution.

STDP rules need to lead to competition and prevent divergent weights. There are a number of strategies to achieve that, such as additive STDP, which leads to strong competition, but needs a hard weight cut off. The weight cut off can be avoided by using multiplicative STDP, which generally leads to weaker competition. A recent alternative, the so called log-STDP rule (Gilson and Fukai 2011) has competition but does not need a weight cut off because it leads to a long-tailed weight distribution. We did not determine explicitly whether log-STDP would lead to anticorrelated networks, but as this is a consequence of the competitive nature of the STDP rule we used, we expect that log-STDP works in the same way, and in addition leads to more realistic log-normal distributions of connection strength.

What could trigger this structural organization in a developing brain? One possibility is that the network restructuring towards anti-correlations occurs after the transition from immature to mature STDP (Itami and Kimura 2012). For the rat somatosensory cortex, this developmental switch coincides with the critical learning period and a period of rapid reorganization of the cortical circuitry (Martens et al. 2015).

Alternatively, in the mature brain plasticity rules could form anti-correlated degrees to obtain cortical circuits sensitive to (nano-)perturbation. After a training period rats respond significantly more to stimulation of a single neuron in the somatosensory cortex than to catch trials, consistent with a sparse cortical code for sensation (Houweling and Brecht 2008; Doron et al. 2014). Thalamic activity that is triggered by whisker stimulation could project preferentially to neurons with high out-degrees. Here, an anti-correlated network configuration could provide simultaneous stability to noise, and sensitivity to (nano)stimulation.

In the networks studied here, synaptic strength was held constant. Consequently, the variability in in-degree results in variable firing rates. For our plasticity experiments, we applied homeostatic scaling such that the network scales towards a specific target firing rate. However, homeostatic scaling could also be applied to individual neurons (Turrigiano 2008). Firing rates of individual neurons converging to a target firing rate could lead to variability in the synaptic strength, thereby reducing the stability of the ACOR network and abolishing the competitive advantage of ACOR compared to PCOR networks.

The recurrent networks were organized without any laminar structure. However, the cortex is organized as a layered structure, generally thought to be comprised of functional cortical columns (Mountcastle 1998; Douglas and Martin 2004), which can improve computational efficiency beyond the capabilities of recurrent networks without such spatial organization (Treves 2003; Raizada and Grossberg 2003; Haeusler and Maass 2007). As we showed here, correlations in the degree distribution can also provide additional capabilities for stimulus detection. Neurons in cortical networks with an anti-correlation in the degree distribution can perform unique roles in the network. The neurons with low in-degree and high out-degree could amplify a signal by projecting to many neurons within a layer or across layers. The neurons with high in-degree and low out-degree could provide improved detection of a network burst by integrating many inputs, and send the detection signal to specific target neurons.

Due to experimental limitations, correlations in the degree distribution have not been directly quantified experimentally (Vasquez et al. 2013). Classical tracing techniques are not appropriate for single neuron studies because they involve connections to or from multiple nearby neurons (Lanciego and Wouterlood 2011). Electron microscopy (EM) based reconstruction of cortical circuitry could provide the complete connectivity structure of a local network. Analyses in a recent review paper (Helmstaedter 2013) show that it could be experimentally feasible to image an appropriately sized block of cortical tissue. However, the main bottleneck is analysis: the detection of synapses and properly identifying the pre- and postsynaptic neuron. The combination of new technologies, such as crowdsourcing (Arganda-Carreras et al. 2015), interactive machine learning (Sommer et al. 2011) and molecular biology (Hirokawa 2011) will make the EM more feasible within a decade. Alternatively, viral based techniques allow crossing of exactly one single synaptic connection which could help to visualize neurons (Wickersham et al. 2007; Osakada et al. 2015). However, to obtain the pre- and postsynaptic connections, single cells should be infected with both anterograde and retrograde crossing viruses, making this a challenging approach. Another method is to simultaneously record from multiple cells and assess connections by inducing action potentials in one neuron at a time and recording the postsynaptic responses in other nearby cells (Song et al. 2005; Perin et al. 2011). Such recordings can be used to estimate the degree distribution indirectly by subsampling. Alternatively, correlations in the degree distribution can be estimated by studying motifs, for example from triplets of neurons (Vasquez et al. 2013). Taken together, we feel that these techniques do not provide a feasible strategy for experimentally confirming our hypothesis on connectivity. We have therefore formulated an alternative approach.

The diversity of interneuron subtypes, generally defined by particular molecular markers such as parvalbumin and somatostatin, have been elegantly interrogated by simultaneous use of molecular, anatomical and electrophysiological techniques on single neurons (Tricoire et al. 2011; Toledo-Rodriguez and Markram 2014). For the excitatory cells here we propose a similar approach: by patch clamping single neurons (1) the electrophysiological profile can be tested, (2) the cell can be colored by dye or virus injection such that the anatomical structure can be reconstructed and (3) by single-cell Reverse Transcriptase-quantative Polymerase Chain Reaction (RT-qPCR) (Freeman et al. 1999), or RNA sequencing (RNA seq) (Zeisel et al. 2015), the mRNA content of the cell can be quantified. The mRNA quantity is an indirect measure of protein expression in the cell. By quantifying the mRNA that code for proteins that are typically found in the presynaptic terminal (such as Neurolexin, Vesicular glutamate transporter 1 and Synaptotagmin-1 Sudhof 2008; Beaudoin et al. 2012; Tang et al. 2006) and proteins that are typically found in the postsynaptic spines (such as Neuroligin, PSD-95, and GluR2 Sudhof 2008; Beaudoin et al. 2012; Dingledine et al. 1999), the in- and out-degree of single neurons can be estimated (Fig. 8B). Thus, by combining the molecular, anatomical and electrophysiological blueprint of the cell’s degree distribution, a (layer-specific) subclassification could be made for single excitatory neurons.

In this study, we showed that correlations in the degree distribution can add computational capabilities for neuronal networks. While intuitively networks that have neurons with high in- and out-degree seem ideal for stimulus detection, we showed that when taking network stability into consideration the detectability was enhanced for networks with anti-correlated degrees. We propose experimental methods to investigate the correlation of in- and out-degree in individual neurons. Furthermore, we have shown how a simple plasticity rule can organize cortical networks to obtain anti-correlations in the degree distribution. Our results suggest that anti-correlation in the degree distribution could be an important strategy to increase stimulus detectability in recurrent cortical networks.

## Electronic supplementary material

Figure S1: Flow of currents in the model. Neurons receive synaptic input (*I*_*syn*_) from other neurons, as indicated by the arrows. All neurons in the model are given white noise (*I*_*fluc*_), and the pyramidal neurons receive in addition background noise spikes (*I*_*bg*_). Stimulation was applied in 1, and up to 6 (default 4) pyramidal neurons, which is indicated by *I*_*stim*_. Sst is short for somatostatin positive neurons, Pyr for pyramidal neurons and PV for parvalbumin positive neurons. (PDF 14.8 KB)

Figure S2: Effect size increases with connection probability. The area-under-curve (AUC) was calculated as before: for each stimulation the network activity was compared to the activity of the network without stimulation. The state of the network variables and random noise generator were identical between the two conditions. An AUC value of 0.5 represents a network that is unable to detect stimulation. For each correlation type the statistics are averaged across 60 networks, error bars are 1 SEM and stars indicate significant differences between ACOR and PCOR networks according to a two-sided t-test. (PDF 14.6 KB)

Figure S3: Effect size increases with network size. The simulated network sizes were: 300, 600 and 1200 neurons, with 80% excitatory and 20% inhibitory neurons and the connection probability was 5%. An AUC value of 0.5 represents a network that is unable to detect stimulation. For each correlation type the statistics are averaged across 60 networks, error bars are 1 SEM and stars indicate significant differences between ACOR and PCOR networks according to a two-sided t-test. (PDF 14.7 KB)

Figure S4: Detection performance is higher for ACOR networks than PCOR networks when the same effective time step was used for u and v. For each correlation type the statistics are averaged across 60 networks, error bars are 1 SEM and stars indicate significant differences between ACOR and PCOR networks according to a two-sided t-test. (PDF 10.0 KB)

Figure S5: Weight-dependent associative plasticity forms networks with anticorrelation in the degree distribution. **A**: A weight-dependent plasticity rule was applied to UCOR networks with 10% connectivity and an upper bound on synaptic strength of 0.013. **B**: At the end of the simulation (100 seconds), a unimodal distribution of synaptic weights was formed. **C**: Pruning of the 5% weakest synapses resulted in anticorrelation in the degree distribution. **D**: Correlation coefficients calculated using the LSR-method were around -1 for the remaining synapses, indicative of ACOR networks. The analysis is based on simulations of 36 different network realizations. (PDF 96.2 KB)
