# Supplementary figures and images for: Anti-correlations in the degree distribution increase stimulus detection performance in noisy spiking neural networks
Source: J Comput Neurosci. 2016 Nov 4;42(1):87–106. doi: 10.1007/s10827-016-0629-1 (PMC5250670; doi:10.1007/s10827-016-0629-1)

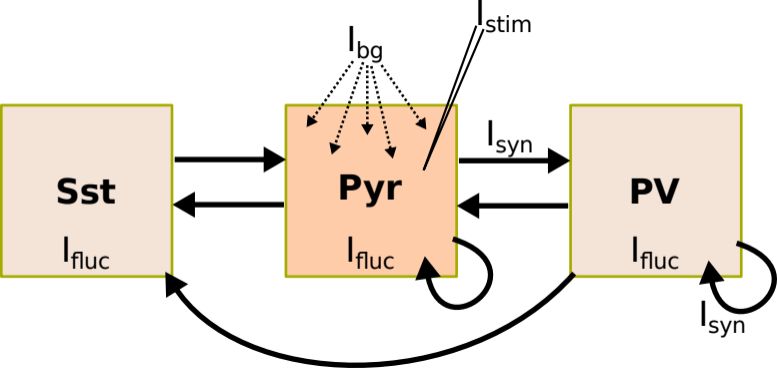

Supplement: Supplementary file 1 — Figure S1: Flow of currents in the model. Neurons receive synaptic input (I syn) from other neurons, as indicated by the arrows. All neurons in the model are given white noise (I fluc), and the pyramidal neurons receive in addition background noise spikes (I bg). Stimulation was applied in 1, and up to 6 (default 4) pyramidal neurons, which is indicated by I stim. Sst is short for somatostatin positive neurons, Pyr for pyramidal neurons and PV for parvalbumin positive neurons. (PDF 14.8 KB) [file 10827_2016_629_MOESM1_ESM.pdf]

probability 1%

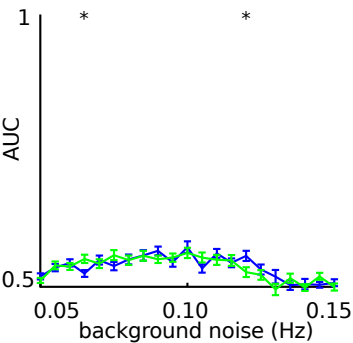

probability 3%

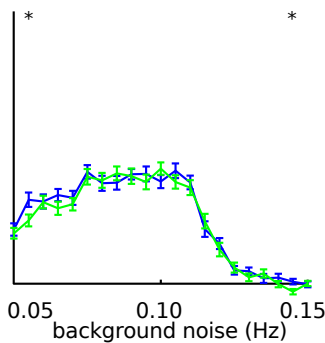

probability 5%

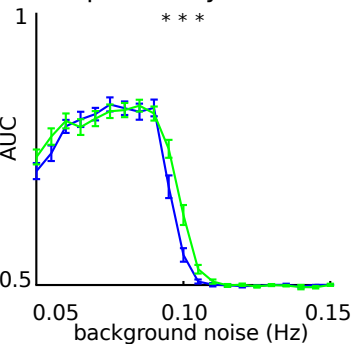

probability 10%

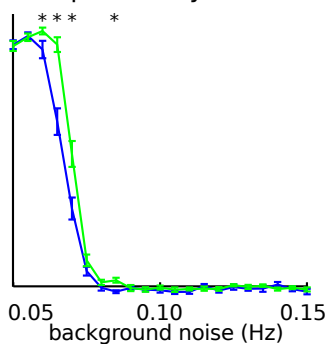

Supplement: Supplementary file 2 — Figure S2: Effect size increases with connection probability. The area-under-curve (AUC) was calculated as before: for each stimulation the network activity was compared to the activity of the network without stimulation. The state of the network variables and random noise generator were identical between the two conditions. An AUC value of 0.5 represents a network that is unable to detect stimulation. For each correlation type the statistics are averaged across 60 networks, error bars are 1 SEM and stars indicate significant differences between ACOR and PCOR networks according to a two-sided t-test. (PDF 14.6 KB) [file 10827_2016_629_MOESM2_ESM.pdf]

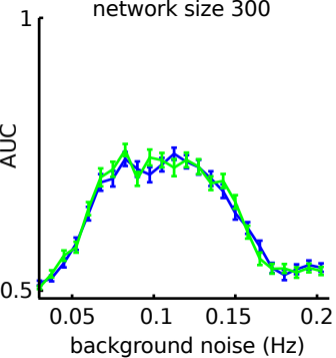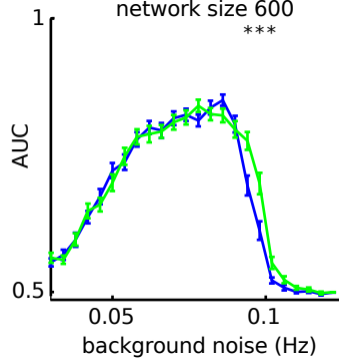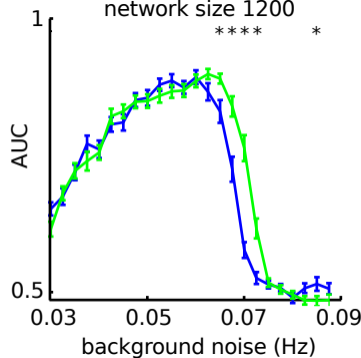

Supplement: Supplementary file 3 — Figure S3: Effect size increases with network size. The simulated network sizes were: 300, 600 and 1200 neurons, with 80% excitatory and 20% inhibitory neurons and the connection probability was 5%. An AUC value of 0.5 represents a network that is unable to detect stimulation. For each correlation type the statistics are averaged across 60 networks, error bars are 1 SEM and stars indicate significant differences between ACOR and PCOR networks according to a two-sided t-test. (PDF 14.7 KB) [file 10827_2016_629_MOESM3_ESM.pdf]

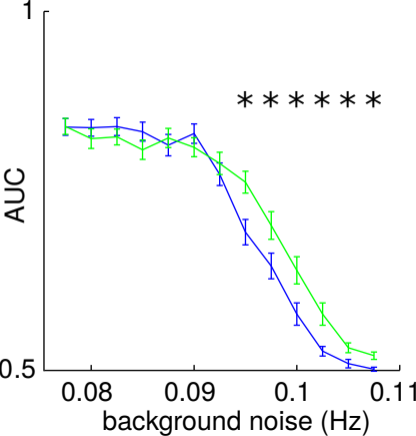

Supplement: Supplementary file 4 — Figure S4: Detection performance is higher for ACOR networks than PCOR networks when the same effective time step was used for u and v. For each correlation type the statistics are averaged across 60 networks, error bars are 1 SEM and stars indicate significant differences between ACOR and PCOR networks according to a two-sided t-test. (PDF 10.0 KB) [file 10827_2016_629_MOESM4_ESM.pdf]

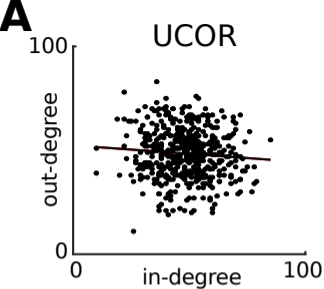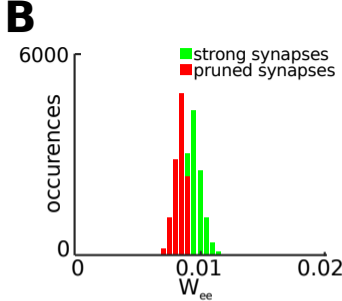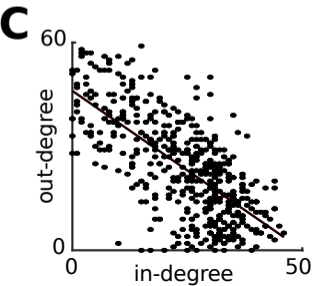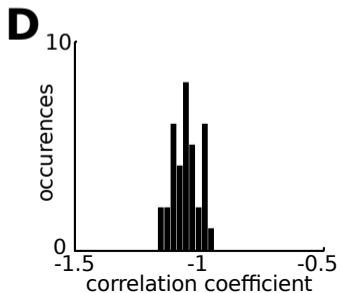

Supplement: Supplementary file 5 — Figure S5: Weight-dependent associative plasticity forms networks with anticorrelation in the degree distribution. A: A weight-dependent plasticity rule was applied to UCOR networks with 10% connectivity and an upper bound on synaptic strength of 0.013. B: At the end of the simulation (100 seconds), a unimodal distribution of synaptic weights was formed. C: Pruning of the 5% weakest synapses resulted in anticorrelation in the degree distribution. D: Correlation coefficients calculated using the LSR-method were around -1 for the remaining synapses, indicative of ACOR networks. The analysis is based on simulations of 36 different network realizations. (PDF 96.2 KB) [file 10827_2016_629_MOESM5_ESM.pdf]
